# Supplementary material for: Mechanistic Insights into the Activation of Lecithin–Cholesterol Acyltransferase in Therapeutic Nanodiscs Composed of Apolipoprotein A-I Mimetic Peptides and Phospholipids
Source: Mol Pharm. 2022 Sep 16;19(11):4135–48. doi: 10.1021/acs.molpharmaceut.2c00540 (PMC9644404; doi:10.1021/acs.molpharmaceut.2c00540)
Supplement: Supplementary file 1 — mp2c00540_si_001.pdf [file mp2c00540_si_001.pdf]

SUPPLEMENTARY INFORMATION
Mechanistic insights into the activation of lecithin-cholesterol acyltransferase in therapeutic nanodiscs composed of apolipoprotein A-I mimetic peptides and phospholipids

Laura Giorgi[1,#], Akseli Niemelä[1,#], Esa-Pekka Kumpula[2], Ossi Natri[1], Petteri Parkkila[3,4], Juha Huiskonen[2], and Artturi Koivuniemi[1,*]

[1]Division of Pharmaceutical Biosciences, Faculty of Pharmacy, University of Helsinki, Finland

[2]Helsinki Institute of Biotechnology, University of Helsinki, Finland

[3]Division of Pharmaceutical Chemistry and Technology, Faculty of Pharmacy, University of Helsinki, Finland

[4]Division of Nano and Biophysics, Department of Physics, Chalmers University of Technology, Sweden

*Correspondence

Artturi Koivuniemi

The Division of Pharmaceutical Biosciences, Faculty of Pharmacy, University of Helsinki, Helsinki, Finland

Tel: +358451217612

E-mail: artturi.koivuniemi@helsinki.fi

[#]The authors contributed equally to the work

SUPPLEMENTARY INFORMATION

Figure S1. Position and orientation of LCAT relative to 22A or 22A-K nanodisc normal based on triplicate 10 μs simulations with LCAT.

Figure S2. Bound 22A-K peptide's angle as a function of distance to other peptides based on extended triplicate 20 μs LCAT open simulations.

Figure S3. Angle as a function of distance between all peptide pairs based on 20 μs simulations without LCAT. Free energy differences to reference position of 1.75 nm and 135° are shown.

Figure S4. Position and orientation of LCAT relative to 22A-R7Q or 22A-K22Q nanodisc normal based on triplicate 10 μs simulations with LCAT.

Figure S5. Contact heat map between peptide residue and all LCAT residues.

Figure S6. Number of dimers per frame.

Table S1. Peptide orientation when bound based on triplicate 10 μs LCAT open simulations. Data as mean (SD of means).

Table S2. Supplementary data on peptide occupancy based on extended triplicate 20 μs LCAT open simulations. Data as mean (SD of means).

Table S3. Configurations of simulated systems.

Table S4. Charged residue-residue contact maps of peptide dimers.

Figure S1

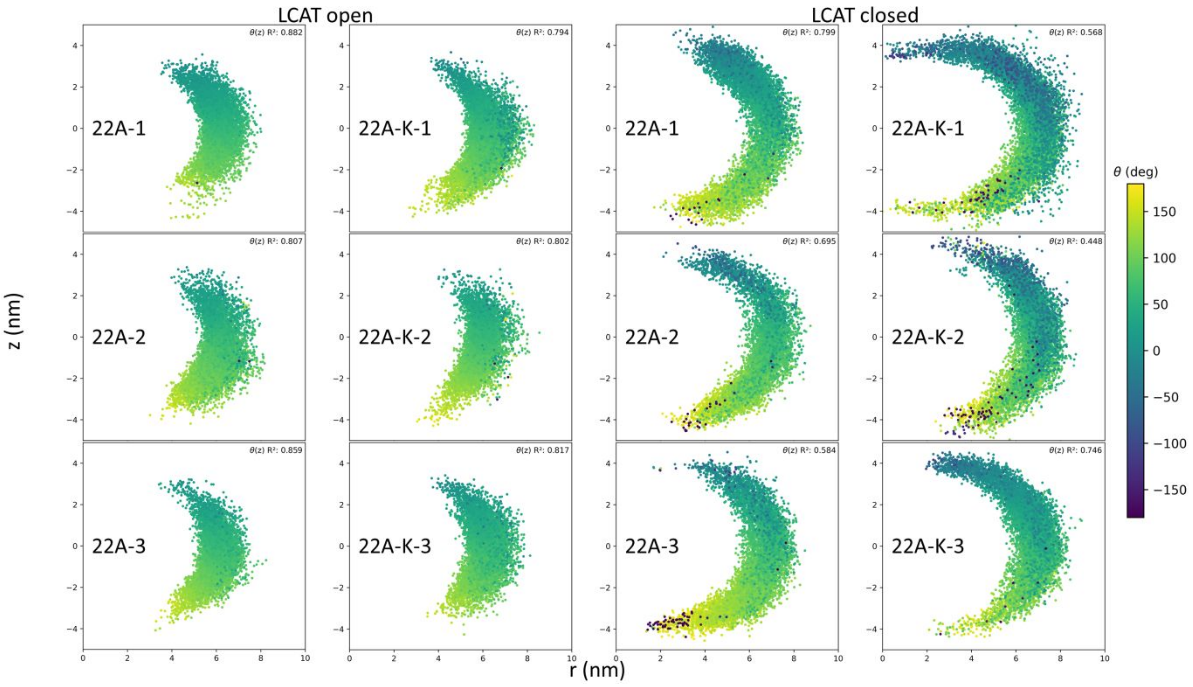

Figure S2

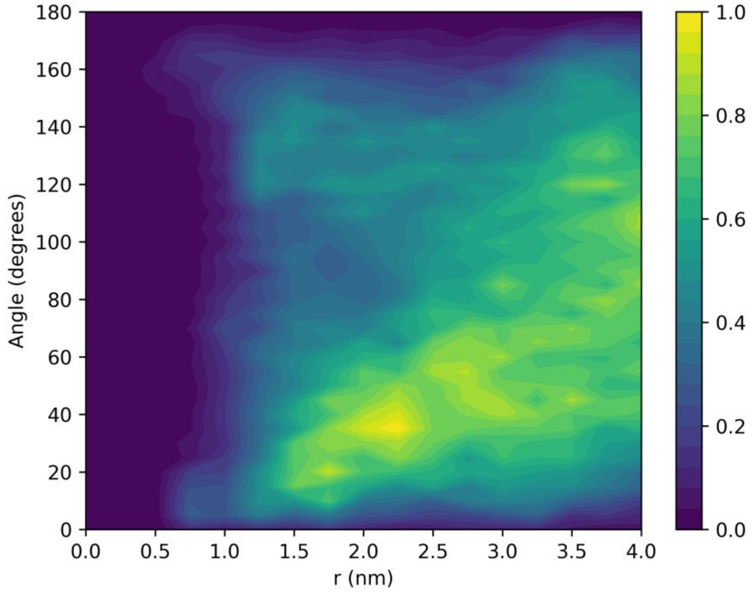

Figure S3

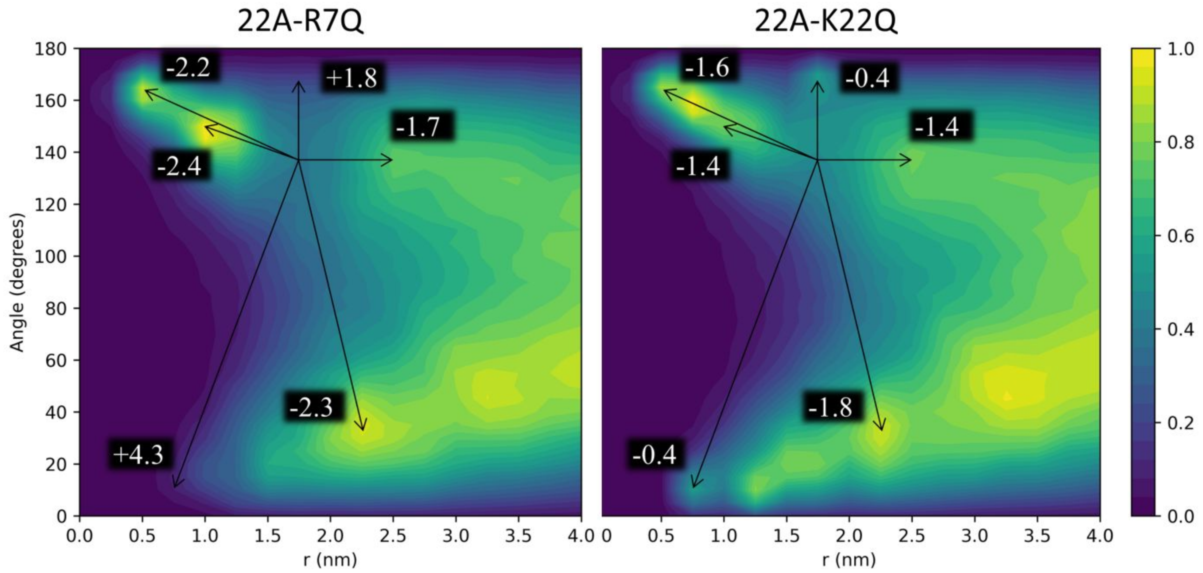

Figure S4

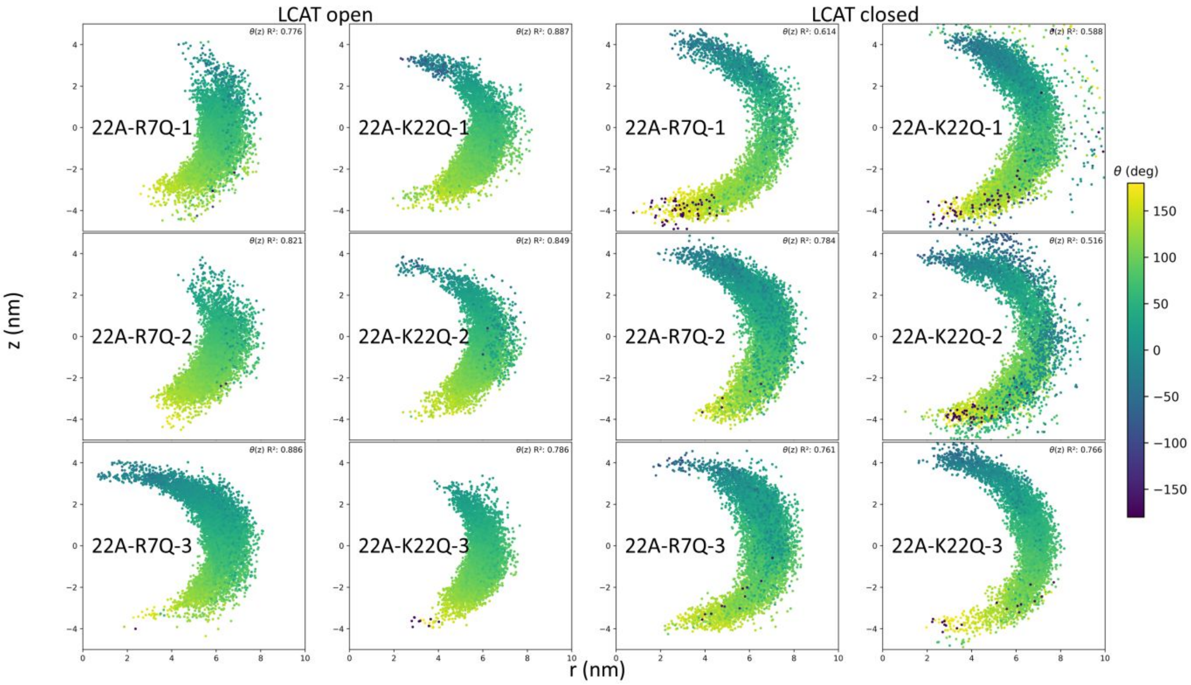

LCAT open
LCAT closed
θ(z) R²: 0.776
22A-R7Q-1
θ(z) R²: 0.887
22A-K22Q-1
θ(z) R²: 0.614
22A-R7Q-1
θ(z) R²: 0.588
22A-K22Q-1
θ(z) R²: 0.821
22A-R7Q-2
θ(z) R²: 0.849
22A-K22Q-2
θ(z) R²: 0.784
22A-R7Q-2
θ(z) R²: 0.516
22A-K22Q-2
θ(z) R²: 0.886
22A-R7Q-3
θ(z) R²: 0.786
22A-K22Q-3
θ(z) R²: 0.761
22A-R7Q-3
θ(z) R²: 0.766
22A-K22Q-3
z (nm)
r (nm)
θ (deg)

Figure S5

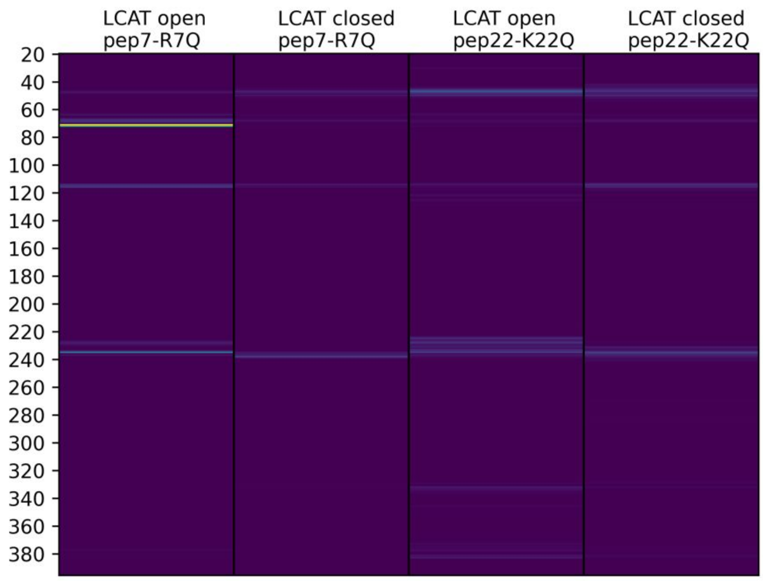

Figure S6

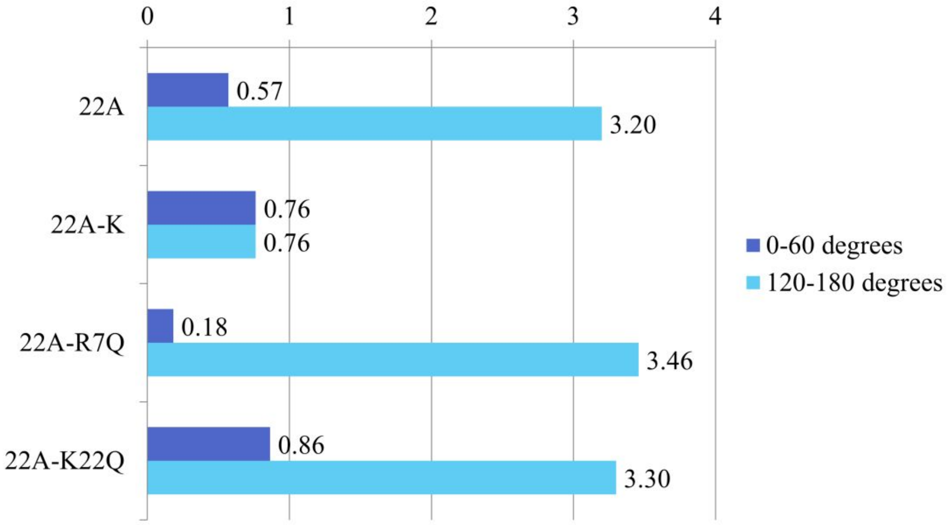

Table S1

Peptide	Peptide vs. CYS31-GLY308 angle (degrees)	Peptide spin (degrees)
22A	45.2(0.8)	2.8(2.8)
22A-K	47.5(0.9)	8.8(1.7)
22A-R7Q	48.7(2.5)	11.4(3.4)
22A-K22Q	43.9(1.1)	4.2(1.0)

Table S2

Peptide	Occupancy (%)	Mean occupancy length (ns)	Max occupancy length (ns)	Num of entry events (n)	Num of peptide changes (n)
22A	31.5(1.8)	4.0(0.1)	83.7(11.4)	1463(118)	6(2.2)
22A-K	17.9(3.2)	3.0(0.1)	42.0(5.9)	1144(165)	10(0.0)
22A-R7Q	32.5(5.4)	4.1(0.5)	71.0(19.4)	1485(85)	5(0.8)

22A-K22Q	24.2(2.6)	3.8(0.3)	62.3(13.6)	1206(188)	5(0.8)

Table S3

Nanodisc configurations
22A+DMPC
22A-K+DMPC
22A-R7Q+DMPC
22A-K22Q+DMPC

System configurations	Length (µs)	Length in effective Martini time (µs)
nanodisc	20	80
nanodisc+LCAT_open-1	10*	40*
nanodisc+LCAT_open-2	10*	40*
nanodisc+LCAT_open-3	10*	40*
nanodisc+LCAT_closed-1	10	40
nanodisc+LCAT_closed-2	10	40
nanodisc+LCAT_closed-3	10	40

*extended until 20 µs (80 µs in effective Martini time) for peptide occupancy analysis

Table S4

0-60 degrees, 0-1.0 nm

22A	PRO1	ASP4	ARG7	GLU8	GLU12	GLU15	LYS18	LYS20	LYS22-TER	LYS22
PRO1	0.00									
ASP4	0.02	0.00								
ARG7	0.01	0.10	0.00							
GLU8	0.00	0.00	0.58	0.00						
GLU12	0.00	0.00	0.49	0.01	0.00					
GLU15	0.00	0.00	0.02	0.00	0.07	0.00				
LYS18	0.00	0.00	0.00	0.03	0.43	0.24	0.00			
LYS20	0.00	0.00	0.00	0.03	0.15	0.32	0.01	0.00		
LYS22-TER	0.00	0.00	0.00	0.00	0.00	0.01	0.24	0.43	0.01	
LYS22	0.00	0.00	0.00	0.00	0.05	0.10	0.01	0.01	0.18	0.00

22A-K	PRO1	ASP4	ARG7	GLU8	GLU12	GLU15	LYS18	LYS20	LEU21-TER
PRO1	0.00								
ASP4	0.01	0.00							
ARG7	0.01	0.09	0.00						
GLU8	0.00	0.00	0.48	0.00					
GLU12	0.00	0.00	0.37	0.02	0.00				
GLU15	0.00	0.00	0.01	0.00	0.06	0.00			
LYS18	0.00	0.00	0.00	0.06	0.43	0.27	0.00		
LYS20	0.00	0.00	0.00	0.01	0.11	0.24	0.01	0.01	
LEU21-TER	0.00	0.00	0.00	0.00	0.00	0.01	0.16	0.54	0.03

22A-R7Q	PRO1	ASP4		GLU8	GLU12	GLU15	LYS18	LYS20	LYS22-TER	LYS22
PRO1	0.00									
ASP4	0.01	0.00								
GLU8	0.00	0.00		0.00						
GLU12	0.00	0.00		0.00	0.00					
GLU15	0.00	0.00		0.00	0.07	0.00				
LYS18	0.00	0.00		0.10	0.67	0.27	0.00			
LYS20	0.00	0.00		0.01	0.05	0.13	0.00	0.01		
LYS22-TER	0.00	0.00		0.00	0.00	0.01	0.10	0.51	0.01	
LYS22	0.00	0.00		0.00	0.17	0.18	0.00	0.01	0.17	0.00

22A-K22Q	PRO1	ASP4	ARG7	GLU8	GLU12	GLU15	LYS18	LYS20	GLN22-TER
PRO1	0.00								
ASP4	0.01	0.00							
ARG7	0.00	0.10	0.00						
GLU8	0.00	0.01	0.66	0.00					
GLU12	0.00	0.00	0.56	0.04	0.00				
GLU15	0.00	0.00	0.02	0.00	0.06	0.00			
LYS18	0.00	0.00	0.00	0.00	0.25	0.28	0.00		
LYS20	0.00	0.00	0.00	0.02	0.11	0.36	0.01	0.00	
GLN22-TER	0.00	0.00	0.00	0.00	0.00	0.00	0.30	0.45	0.01

120-180 degrees, 0-1.0 nm

22A	PRO1	ASP4	ARG7	GLU8	GLU12	GLU15	LYS18	LYS20	LYS22-TER	LYS22
PRO1	0.00									
ASP4	0.00	0.00								
ARG7	0.00	0.00	0.00							
GLU8	0.00	0.00	0.00	0.00						
GLU12	0.00	0.00	0.00	0.00	0.02					
GLU15	0.00	0.00	0.02	0.00	0.00	0.01				
LYS18	0.00	0.01	0.02	0.27	0.03	0.28	0.00			
LYS20	0.00	0.21	0.00	0.95	0.87	0.09	0.00	0.00		
LYS22-TER	0.03	0.01	0.48	0.01	0.01	0.00	0.00	0.00	0.00	
LYS22	0.01	0.20	0.02	0.24	0.04	0.00	0.00	0.00	0.00	0.00

22A-K	PRO1	ASP4	ARG7	GLU8	GLU12	GLU15	LYS18	LYS20	LEU21-TER
PRO1	0.00								
ASP4	0.00	0.00							
ARG7	0.00	0.00	0.00						
GLU8	0.00	0.00	0.00	0.00					
GLU12	0.00	0.00	0.00	0.00	0.01				
GLU15	0.00	0.00	0.02	0.00	0.00	0.05			
LYS18	0.00	0.02	0.01	0.35	0.39	0.62	0.01		
LYS20	0.00	0.13	0.02	0.65	0.61	0.07	0.00	0.00	
LEU21-TER	0.03	0.01	0.49	0.04	0.03	0.00	0.00	0.00	0.00

22A-R7Q	PRO1	ASP4		GLU8	GLU12	GLU15	LYS18	LYS20	LYS22-TER	LYS22
PRO1	0.00									
ASP4	0.00	0.00								
GLU8	0.00	0.00		0.00						
GLU12	0.00	0.00		0.00	0.03					
GLU15	0.00	0.00		0.00	0.00	0.01				
LYS18	0.00	0.00		0.15	0.02	0.15	0.00			
LYS20	0.00	0.36		1.22	0.98	0.10	0.00	0.00		
LYS22-TER	0.04	0.01		0.01	0.00	0.00	0.00	0.00	0.00	
LYS22	0.01	0.23		0.27	0.04	0.00	0.00	0.00	0.00	0.00

22A-K22Q	PRO1	ASP4	ARG7	GLU8	GLU12	GLU15	LYS18	LYS20	GLN22-TER
PRO1	0.00								
ASP4	0.00	0.00							
ARG7	0.00	0.00	0.00						
GLU8	0.00	0.00	0.00	0.00					
GLU12	0.00	0.00	0.00	0.00	0.02				
GLU15	0.00	0.00	0.02	0.00	0.00	0.01			
LYS18	0.00	0.01	0.01	0.31	0.03	0.27	0.00		
LYS20	0.00	0.32	0.00	0.98	0.69	0.04	0.00	0.00	
GLN22-TER	0.04	0.01	0.52	0.01	0.00	0.00	0.00	0.00	0.00
